# Macrophage colony‐stimulating factor as a weapon against cytomegalovirus

**DOI:** 10.15252/emmm.202318319

**Published:** 2023-09-12

**Authors:** Eric Solary

**Affiliations:** ^1^ INSERM UMR 1287 and Department of Hematology Gustave Roussy Villejuif France; ^2^ Faculty of Medicine Université Paris‐Saclay Le Kremlin‐Bicêtre France

**Keywords:** Immunology, Stem Cells & Regenerative Medicine

## Abstract

Cytomegalovirus (CMV) infection is one of the severe opportunistic infections faced by severely immunocompromised patients. High viral loads cause tissue‐invasive disease and expose to death or various indirect effects. Substantial progress was made in monitoring active infection, and antiviral drugs were developed. However, dose‐limiting toxicities and genotypic resistance limit therapeutic efficacy and vaccine development is hampered by the complex biology of the virus. In this issue of *EMBO Molecular Medicine*, Kandalla *et al* (2023) suggest an innovative strategy using the cytokine macrophage colony‐stimulating factor (M‐CSF) whose clinical development was left behind two decades ago. By stimulating an endogenous immune defense mechanism, M‐CSF promotes viral clearance in a mouse model of hematopoietic stem cell transplantation, without impairing stem cell engraftment. These results reactivate the interest in the potential therapeutic use of this cytokine.

## Graphical abstract

E. Solary discusses potential therapeutic use of macrophage colony‐stimulating factor against cytomegalovirus in immunocompromised individuals as reported by M. Sieweke and colleagues, in this issue of *EMBO Mol Med*.

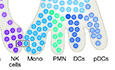

Human CMV, a double‐strand DNA virus of the herpes family, is a ubiquitous pathogen that is usually acquired in childhood or early adult life through contact with infected individuals' body fluids. About 70% of the population across the world carry evidence of infection. Primary CMV infection in immunocompetent people is usually asymptomatic or induces mild clinical manifestations. The virus subsequently persists lifelong in a latent form in myeloid cells of the host. Its reactivation and propagation is a common opportunistic situation in immunocompromised patients, including hematopoietic stem cell and solid organ transplantation recipients, patients with human immunodeficiency virus infection, and those on immunosuppressive therapy. In these patients, CMV infection can become severe and invasive, increases the rate of bacterial or fungal infections, promotes graft rejection or dysfunction by interfering with immunological tolerance, and eventually exposes to death (Griffiths & Reeves, 2021). Only a few antiviral medications are approved for the prevention or treatment of CMV infection, the most common being oral valganciclovir, but genotypic drug resistance and dose‐limiting toxicities decrease their efficacy. The development of a vaccine against CMV is considered the highest priority but remains challenging due to the complex biology of the virus, and alternate oligonucleotide‐based antiviral strategies are in preclinical stage (Panda *et al*, 2023). In this issue of *EMBO Molecular Medicine*, Kandalla *et al* (2023) explore another approach. In a mouse model of hematopoietic stem cell transplantation, these authors demonstrate that the cytokine macrophage colony‐stimulating factor (M‐CSF; also known as CSF‐1) protects from lethal murine CMV infection through the activation of a coordinated myeloid‐natural killer (NK) cell differentiation program that reconstitutes antiviral activity, leading to viral clearance without impairing stem cell engraftment. The originality of this approach is to stimulate an endogenous immune defense mechanism rather than targeting the virus itself, bridging a gap between preventive vaccine and antiviral drugs.

While recombinant human G‐CSF is routinely used in clinics to stimulate the recovery of granulocytes after myelosuppressive therapy, there was no reported follow‐up from the initial clinical trials testing M‐CSF in humans (Hume & MacDonald, 2012). Yet, a striking difference between the two cytokines is that G‐CSF acts on late progenitor cells to promote their maturation while M‐CSF directly engages the production of innate immune cells by instructing commitment of hematopoietic stem and early progenitor cells (Mossadegh‐Keller *et al*, 2013), suggesting that it could shorten the period of therapy‐induced myelodepletion. Accordingly, the authors previously demonstrated a protective effect of M‐CSF, but not G‐CSF, against lethal infection with the bacteria *Pseudomonas aeruginosa* and the fungus *Aspergillus fumigatus* in transplanted mice. Importantly, M‐CSF stimulates the production of diverse immune cells (Kandalla *et al*, 2016), suggesting that the cytokine could protect against a broad spectrum of infectious agents, potentially including viruses. This antiviral effect is now validated in mice.

The merit of the study by Kandalla *et al* (2023) is to decipher the mechanisms of the antiviral response triggered by M‐CSF. Two pillars are needed to get a full‐blown effect of the cytokine. One is myelopoiesis, including the generation of monocytes, granulocytes, conventional dendritic cells, and plasmacytoid dendritic cells (pDCs), whose importance is nicely validated by a combination of loss‐ and gain‐of‐function approaches. Importantly, M‐CSF promotes the production of type I interferon, a first‐line antiviral cytokine, by pDCs and monocytes. The second one is an increase in the number and activity of donor‐derived progenitor and mature NK cells whose antibody‐mediated depletion abolishes M‐CSF effect (Fig 1A). Importantly, an increased number and activity of NK cells were previously detected in human patients receiving M‐CSF (under the name Mirimostim) to correct severe myelosuppression induced by chemotherapy for ovarian cancer (Hidaka *et al*, 2003), suggesting that M‐CSF reproduces in humans the effect depicted in mice.

**Figure 1:**
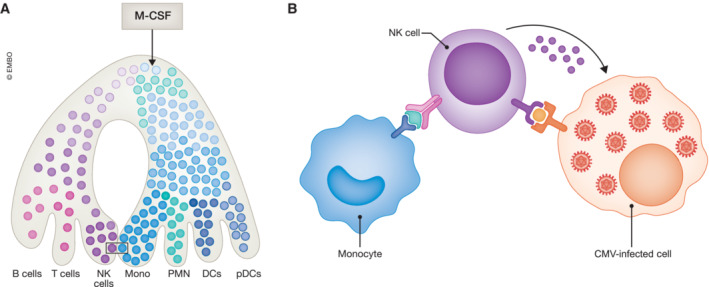
M‐CSF promotes myelopoiesis and a monocyte‐natural killer (NK) cell program to eliminate CMV‐infected cells (A) M‐CSF was shown to instruct a fraction of hematopoietic stem and progenitor cells (light yellow) in the bone marrow to promote myelopoiesis through a stepwise differentiation process generating monocytes, polymorphonuclear cells (PMN), conventional dendritic cells (cDC), and type I interferon producing plasmacytoid dendritic cells (pDCs), without preventing the generation of other lineages; (B) interaction between monocytes and NK cells: IL5Rα at the surface of monocytes ensures transpresentation of IL‐15 (in green) to NK cells expressing IL15‐Rβ (light beige) and γc (dark beige), promoting NK cell maturation through NKP and their activation. In turn, NK cells may kill virus‐infected cells through cell–cell contact and the release of toxic proteins such as perforin.

One of the key cytokines involved in the final maturation and survival of NK cells is interleukin‐15 (IL‐15). At steady state, low levels of IL‐15 are expressed, mainly by myeloid cells, with monocytes playing a key role in NK cell development and homeostasis. IL‐15 functions through a delivery process termed transpresentation in which IL‐15‐expressing cells simultaneously express IL‐15Rα to supply IL‐15 to IL‐15‐responsive NK cells. Kandalla *et al* demonstrate that M‐CSF increases *IL15* mRNA, *IL15RA* mRNA, and IL‐15Rα protein expression, mostly on Ly6C^high^ monocyte subset, and that transfer of granulomonocyte progenitors (GMP) from *IL15RA* knockout mice fail to reproduce the protective effect of wild‐type GMPs in the studied model. Interestingly, type I interferon produced upon M‐CSF treatment contributes to increasing IL‐15 production by myeloid cells (Fig 1B). Together, IL‐15 is a link between the two pillars of M‐CSF protective effect. The question remains whether M‐CSF also promotes the production of cytokines that drive early stages of immune lymphoid cell differentiation.

In addition to elegantly decipher some of the key mechanisms involved in M‐CSF protective activity toward lethal effects of CMV infection, Kandalla *et al* demonstrate that, in the studied model, M‐CSF does not preclude bone marrow engraftment nor it promotes graft‐versus‐host disease, defusing a potentially controversial issue. Nevertheless, clinical development of M‐CSF to treat severe CMV infection in immunocompromised patient will require careful monitoring of other potentially negative effects. CSF‐1R, the surface receptor that binds M‐CSF, is expressed by some leukemia cells (Romine *et al*, 2021; Simonis *et al*, 2021), while CSF‐1R‐expressing resident macrophages in bone marrow are requested for the persistent engraftment of long‐term reconstituting hematopoietic stem cells (Kaur *et al*, 2018). Therefore, M‐CSF may be used on a short term not to promote the growth of residual leukemic cells after transplantation nor to compromise cell engraftment. M‐CSF may also be tested carefully in solid tumor patients with CMV infection as tumor‐associated macrophages (TAM) express CSF‐1R (Wen *et al*, 2023). With these caveats in mind, reactivating the clinical development of this cytokine, especially in the context of severe CMV infection, certainly deserves further consideration.
